# Classification of micro-calcification in mammograms using scalable linear Fisher discriminant analysis

**DOI:** 10.1007/s11517-017-1774-z

**Published:** 2018-01-25

**Authors:** Zobia Suhail, Erika R. E. Denton, Reyer Zwiggelaar

**Affiliations:** 1 Aberystwyth University, Aberystwyth, UK; 2 Norfolk and Norwich University Hospital, Norwich, UK

**Keywords:** Micro-calcification, Classification, Fisher discriminant analysis, Principal component analysis, Computer aided detection, Dimensionality reduction

## Abstract

Breast cancer is one of the major causes of death in women. Computer Aided Diagnosis (CAD) systems are being developed to assist radiologists in early diagnosis. Micro-calcifications can be an early symptom of breast cancer. Besides detection, classification of micro-calcification as benign or malignant is essential in a complete CAD system. We have developed a novel method for the classification of benign and malignant micro-calcification using an improved Fisher Linear Discriminant Analysis (LDA) approach for the linear transformation of segmented micro-calcification data in combination with a Support Vector Machine (SVM) variant to classify between the two classes. The results indicate an average accuracy equal to 96% which is comparable to state-of-the art methods in the literature.

**Graphical Abstract:**
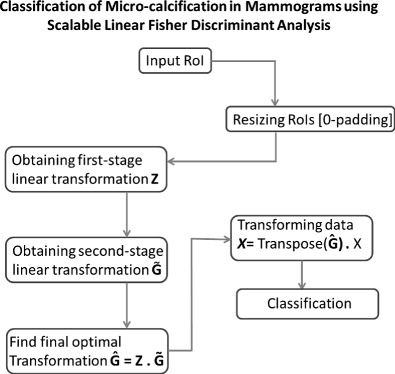
Classification of Micro-calcification in Mammograms using Scalable Linear Fisher Discriminant Analysis

Classification of Micro-calcification in Mammograms using Scalable Linear Fisher Discriminant Analysis

## Background

Machine Learning is widely being used to solve problems involving high dimensional data. In a large number of cases, the dimension of the data is much larger than the sample size, which is referred to as the undersampling problem [1]. High dimesionality and undersampling occurs in many applications [2, 3]. One of the solutions to deal with this undersampling problem is dimensionality reduction [3, 4].

Principal component analysis (PCA) is a procedure to convert a number of correlated variables into fewer variables called principal components [5], commonly used in fields of pattern recognition and computer vision [6, 7]. The purpose of PCA is to transform data to some low dimensional space and subsequently apply a classification method. Fisher Linear Discriminant Analysis (LDA) has been around for a long time with applications found in face recognition [3, 8], marketing [9], and biomedical studies [10]. LDA is a classical approach used for feature extraction and dimensionality reduction [11, 12]. The objective function of conventional LDA is to find a linear transformation, where the class separation is maximized while keeping the in-class variance small [5]. One of the major problems associated with LDA in the singularity issue [13]: LDA requires the scatter matrices of the training data to be non singular but the training samples are from a high dimensional space and in most cases the sample size is smaller than this dimension leading to a sparse matrices and potential singularity issues. Many LDA extensions have been proposed to resolve this singularity issue. PCA + LDA [3] and LDA/QR [4] are some of these two-stage extensions. The purpose of the two-stage approach is to convert the data into some intermediate form before applying the actual LDA. While applying these two-stage algorithms some of the important information may be lost in the first dimesionality reduction stage that may be beneficial for the subsequent LDA [14].

Zhang et al. [14] proposed a fast two-stage LDA algorithm as an alternative to the PCA+LDA or QR/LDA solutions. They claimed with the theoretical analysis that their algorithm outfperforms the other two-staged algorithms [3, 4] with the same scalability. They also provided the theoretical bound on the approximation of two-staged LDA. We propose a novel application of the scalable-LDA approach [14] for the classification of breast calcifications. To our knowledge, such method of feature extraction through dimensionality reduction has never been used for the problem of classifying micro-calcifications. The proposed method provides a way of encoding binary calcification data to a single value, which is a one-dimensional representation of the high dimension micro-calcification data. Instead of using a large number of features, the proposed classification approach used only a single feature to distinguish between the benign and malignant micro-calcifications. In terms of classification accuracy, the algorithm is giving good results compared to other state-of-the-art approaches developed for the classification of malignant and benign micro-calcifications (an overview of state-of-the-art approaches developed for the classification of benign and malignant micro-calcification as well as comparison with the current method can be found in Section 6.1).

## Dataset

**Fig. 1:**
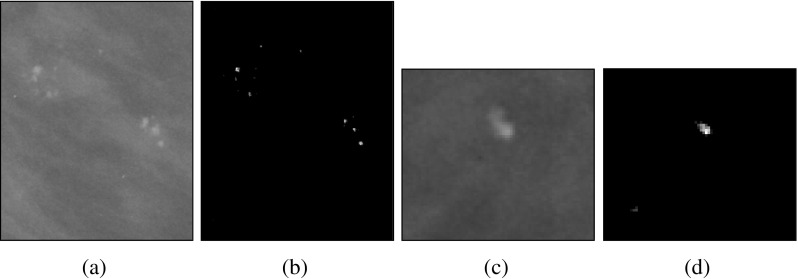
Sample RoIs from the benign class: **a** and **c** are showing original mammogrphic RoIs, whereas **b** and **d** are showing the segmented micro-calcifications

**Fig. 2:**
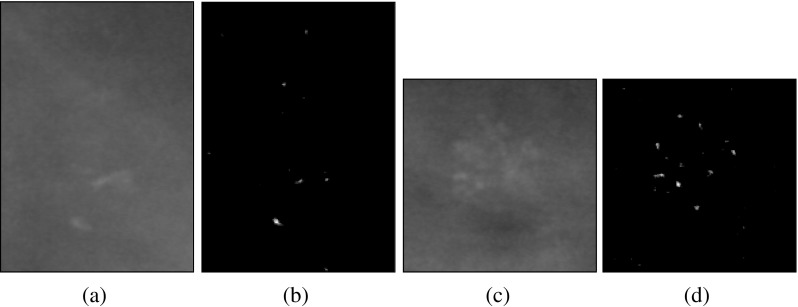
Sample RoIs from the malignant class: **a** and **c** are showing original mammographic RoIs, whereas **b** and **d** are showing the segmented micro-calcifications

We used data from the Digital Database for Screening Mammography (DDSM) [15], which contains identified micro-calcifications. The mammograms in the DDSM database were digitized by one of four scanners: DBA M2100 ImageClear (42 *μ* m per pixel, 16 bits), Howtek 960 (43.5 *μ* m per pixel, 12 bits), Lumisys 200 Laser (50 *μ* m per pixel, 12 bits), and Howtek MultiRad850 (43.5 *μ* m per pixel, 12 bits). The patches are extracted from the whole mammogram containing micro-calcifications according to the available information regarding position of micro-calcification clusters. In addition, we excluded cases with overlapping mass regions. Subsequently, the micro-calcification clusters are either automatically detected [16] or manually annotated by expert radiologists. The automatic approach for segmenting micro-calcification clusters [16] involves local feature extraction using filter banks (including delta, Gaussian and Laplacian filters). After performing training to select salient features, a boosting classifier is used to detect individual micro-calcifications. Segmented images are representing the micro-calcification in binary form, where 0’s indicate the absence of micro-calcifications and 1’s the presence of micro-calcifications. The images in this dataset are of variable sizes. The average image size is (482 × 450), whereas the maximum height of all the images from the dataset is 2754 and the maximum width is 3778. The dataset has in total 288 ROIs (139 malignant and 149 benign). Each of the 288 RoI belongs to a different woman. Some sample variations in the size of the RoIs used in this study can be seen in Figs. 1 and 2 covering benign and malignant classes, respectively.

## Fisher discriminant analysis

Given a data matrix $D=[d_{1}, d_{2},....d_{n}]^{T} \in \mathbb {R}^{n\times m}$, where d_*i*_ is a m-dimensional vector, let the data matrix *D* be partitioned into *k* classes as *D*^*T*^ = [*D*1*T*,*D*2*T*,.....*DkT*], where $D_{i} \in \mathbb {R}^{n_{i}\times m}$ and ${\sum }_{i = 1}^{k} n_{i} = n$. The objective of conventional LDA is to compute the optimal linear transformation $G \in \mathbb {R}^{m\times l}$ such that the class structure of the original space is preserved in the low-dimensional space. So, G maps each *d*_*i*_ of *D* in the *m*-dimensional space to a vector *y*_*j*_ in *l*-dimensional space.

$$G:d_{i} \in \mathbb{R}^{m} \rightarrow y_{j}=G^{T}d_{i} \in \mathbb{R}^{l} (l<m) $$

For discriminant analysis [12], two scatter matrices (between class and total scatter matrices) are defined as:

1 $$ S_{b} \,=\, \frac{1}{n} \!\sum\limits_{i = 1}^{k} \!\underset{d \in D_{i}}{\sum} \!(c_{i}\,-\,c)(c_{i}\!-{\kern-.5pt}c)^{T} = \frac{1}{n} \!\sum\limits_{i = 1}^{k} n_{i} (c_{i}-c)(c_{i}-c)^{T}  $$

2 $$ S_{t} = \frac{1}{n} \sum\limits_{i = 1}^{n} (d_{i}-c)(d_{i}-c)^{T}  $$

where $c_{i} = \frac {1}{n_{i}} {\sum }_{d \in D_{i}} d $ is the mean of the *i*^*th*^ class and $c=\frac {1}{n} {\sum }_{d \in D} d $ is the mean of the whole data set. In the low-dimensional space, obtained as a result of linear transformation *G*, the scatter matrices become:

$${S_{b}}^{L} = G^{T} S_{b} G, {S_{t}}^{L} = G^{T} S_{t} G $$

The calculation of the scatter matrices can be simplified through using precursors *H*_*b*_ and *H*_*t*_ as:

3 $$ H_{b}= \frac{1}{\sqrt{n}} (\sqrt{n_{1}}(c_{1} - c) {\ldots} \sqrt{n_{k}}(c_{k} - c))  $$

4 $$ H_{t}=\frac{1}{\sqrt{n}} ({D}^{T} - c{e}^{T})  $$

where $e = [1,...,1]^ T \in \mathbb {R}^{n}$, then the scatter matrices *S*_*b*_ and *S*_*t*_ can be expressed as:

$$S_{b} = H_{b}{H_{b}}^{T}, S_{t}=H_{t} {H_{t}}^{T} $$

An optimal transformation G can be obtained by using the optimization from classical discriminant analysis [12]:

$$\underset{G}{\arg\max} \{trace (({S}_{t}^{L})^{-1} {S}_{b}^{L})\} $$

The solution to this optimization can be obtained by applying eigen-decomposition on the matrix *S*_*t*_^− 1^*S*_*b*_, if *S*_*t*_ is non-singular [12]. However, if *S*_*t*_ is singular, we can use the eigen-decomposition of *St*‡*S*_*b*_, where *St*‡ is the pseudo-inverse of *S*_*t*_. The use of psuedo-inverse for LDA has been studied in the literature [17, 18]. Ye [19] proposed a SVD-based solution for the eigen-decomposition (Algorithm 1), according to which the optimal transformation are the top q eigenvectors of *St*‡*S*_*b*_, where q is equal to rank (*H*_*b*_), which in most cases is equal to *k* − 1 (k is the number of classes). Zhang et al. [14] proposed a two-stage LDA algorithm as a scalable version of the conventional LDA. At the first stage of their algorithm, they introduced a linear transformation $Z \in \mathbb {R}^{m \times r}$ to reduce the data dimensionality to some intermediate dimension *r*, and then applied conventional LDA on the reduced total scatter matrix $\tilde {S_{t}}=Z^{T} S_{t} Z$ and reduced between-class scatter matrix $\tilde {S_{b}} = Z^{T} S_{b} Z$ (by representing them as precursors $\tilde {H_{b}}$ and $\tilde {H_{t}}$) in order to get the linear transformation $\tilde {G}$. In the final stage they produced the reduced transformation as $\hat {G} = Z \tilde {G}$. The pseudocode of this approach is presented in Algorithm 2. 
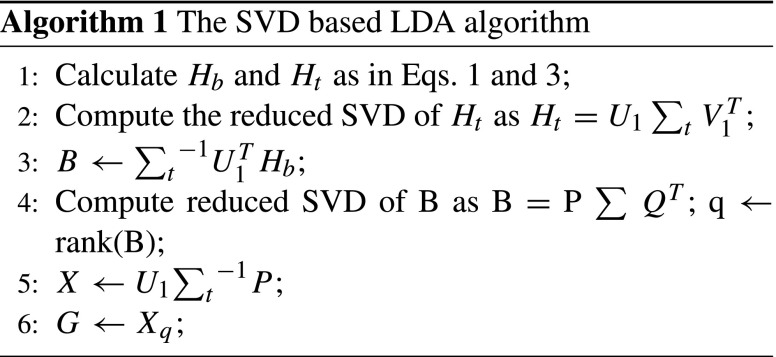
 
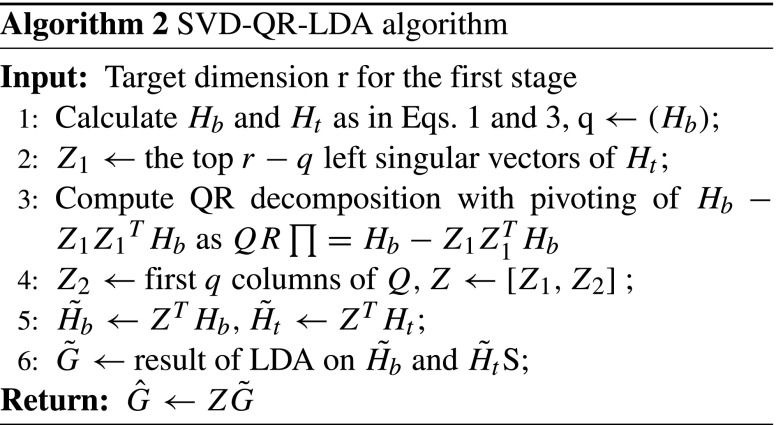


## Scalable LDA

**Fig. 3:**
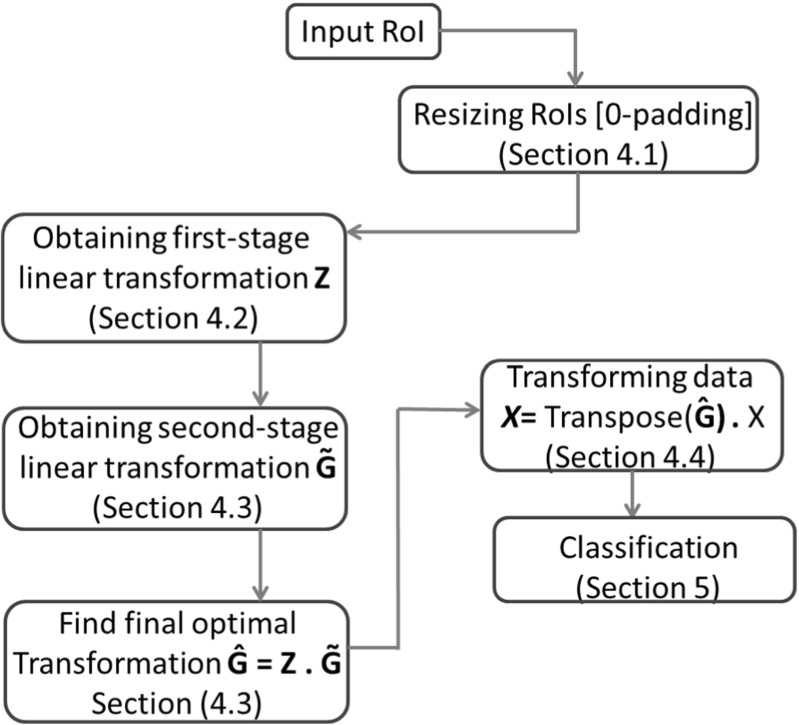
Process flow of the proposed method

In this section, we will explain the overall experimental setup for the Scalable-LDA approach implementation on the given dataset of segmented micro-calcifications with the aim of classifying them as benign or malignant micro-calcifications. The flow of the whole process can be seen in Fig. 3.

### Resizing the RoIs

As explained in Section 2, we have 288 RoIs from the DDSM database. The basic purpose of this research was to devise a way to distinguish between the two classes of micro-calcification (i.e., benign versus malignant). Algorithm 2 required for all images to be equal size. For this, we resized all the images to max-height × max-width so that we are not loosing any single piece of information from any image within the dataset. We did this resizing by retaining the original image data I(x,y) at the center and fill the extra max-height-*I*_*x*_ and max-width-*I*_*y*_ pixels with the value 0. The purpose of using the proposed 0-padding technique instead of any other resizing approach (e.g. interpolation) is to retain the original data. The resizing through this 0-padding will keep the data at the centre of the image frame without adding additional bits to the original data or removing image information. After resizing the images in the dataset, all the images have size equal to 2754 × 3778.

### Getting the intermediate dimensions [two-stage LDA]

Subsequently, we vectorize each image by converting it from 2754 × 3778 to 10404612 × 1 dimensions. In order to apply Fisher LDA for the dataset with this large dimensionality, we need to reduce the dimensionality of the data. As explained in Section 2, we have 149 images from the benign class and 139 from the malignant class, so we have 149 and 139 feature vectors from the benign and malignant classes. Here, we used terms *D*_*benign*_ to represent the data belonging to the benign class and *D*_*malignant*_ represents data from the malignant class, which are:

$$D_{benign}=[d_{1}, d_{2},....d_{q}]^{T} \in \mathbb{R}^{q\times m}, q = 149, \text{ and} $$

$$D_{malignant}=[d_{q + 1}, d_{q + 2},....d_{q+l}]^{T} \in \mathbb{R}^{l\times m}, l = 139. $$

The total dataset is then represented as a data matrix *D*^*T*^ as [*DbenignT*,*DmalignantT*], where the total size of *D*_*benign*_ is 149 × 10404612 and 139 × 10404612 for *D*_*malignant*_ according to the size of the dataset for each class. The total size of the final data matrix *D* is 288 × 10404612 (containing data vectors from both benign and malignant classes). As the sample size is much less than the dimensionality of the feature space, we can not apply the conventional LDA to solve this problem. To execute Algorithm 2, we need to compute the the precursors *H*_*b*_ and *H*_*t*_ (Eqs. 3 and 4) which requires the mean of the two classes. These precursors i.e. *H*_*b*_ and *H*_*t*_, are required by Algorithms 1 and 2 to obtain the intermediate and final linear transformations.

We compute the mean for dataset *D*_*benign*_, *D*_*malignant*_ and D as:

$$c_{benign} = \frac{1}{q} {\sum}_{d \in D_{benign}} d $$

$$c_{malignant} = \frac{1}{l} {\sum}_{d \in D_{malignant}} d \,\,\text{ and} $$

$$c_{total} = \frac{1}{q+l} {\sum}_{d \in D} d. $$

By using means *c*_*benign*_, *c*_*malignant*_ and *c*_*total*_, we compute the precursors through Eqs. 1 and 2. We run Algorithm 2 by setting the value of *r*= 23 for the first stage (details of finding optimal value of *r* can be found in Section 5). For Algorithm 2 to perform well, the value of *r*≫ q [14]and a value to 23 is much larger than *q* (*q*= 1 for our data, as *q*= number of classes − 1̇). After executing step 1–4, we obtained the linear transformation $Z \in \mathbb {R}^{10404612 \times 23}$, to reduce the dimensionality to 23.

### Final linear transformation [two-stage LDA]

After obtaining the intermediate linear transformation *Z*, the next step is to find the final optimal transformation. Subsequently, after executing step 5 of Algorithm 2, we obtained $\tilde {H_{b}}$ and $\tilde {H_{t}}$ (having dimensionality (23 × 2) and (23 × 288), respectively) which will be used in step 6 of Algorithm 2, where we used the method proposed in Algorithm 1 for performing LDA (using precursors $\tilde {H_{b}}$ and $\tilde {H_{t}}$) instead of using conventional LDA. The result of this LDA operation at step 6 of Algorithm 2 is a linear transformation $\tilde {G}$ having dimensionality 23 × 1. Whereas the dimensionality of transformation matrix *Z* at step 4 is (10404612 × 23).

After getting the linear transformations matrix *Z* and $\tilde {G}$, the next step is to obtain the final optimal transformation $\hat {G}$, which is computed by multiplying the transformation matrices *Z* (10404612 × 23) and $\tilde {G}$ (23 × 1). The dimensionality of this final optimal transformation matrix is (10404612 × 1).

### Transforming the data to the reduced dimension

Next we transform our data (being represented as a vector [1 × 10404612]^*T*^) using the linear transformation $\hat {G}$. We get the transformation as $\hat {G}^{T} d_{i}$ ,where *d*_*i*_ ∈ *d*_1_,*d*_2_…*d*_*q*_,*d*_*q*+ 1_,*d*_*q*+ 2_…*d*_*q* + *l*_, where $\hat {G}$ transformed each vector d from D to a one-dimensional space. The result of this projection is a single value for each data vector. The next step is to verify and check whether the data is linearly separable or not, and to use this to classify the data into the benign and malignant classes.

## Performance evaluation

We classified our projected data with five different classifiers, which are Support Vector Machine (SVM), Baysian Network (BN), K-Nearest Neightbour (K-NN), Decision Table (DT) and ADTree. We used Weka [20] for all these experiments (developer version 3.7.2) and we left all the classifier’s parameters at the default setting.

As the projected data is one-dimensional, one of the ways to classify the data is to use a Support Vector Machine (SVM) [5]. We used Sequential Minimal Optimization (SMO) which is available in Weka [20] as an efficient way to solve the SVM problem [21]. We used a 10 runs 10-fold cross-validation (10-FCV) scheme for the performance evaluation of our results.

### Optimizing the parameter *r*

**Fig. 4:**
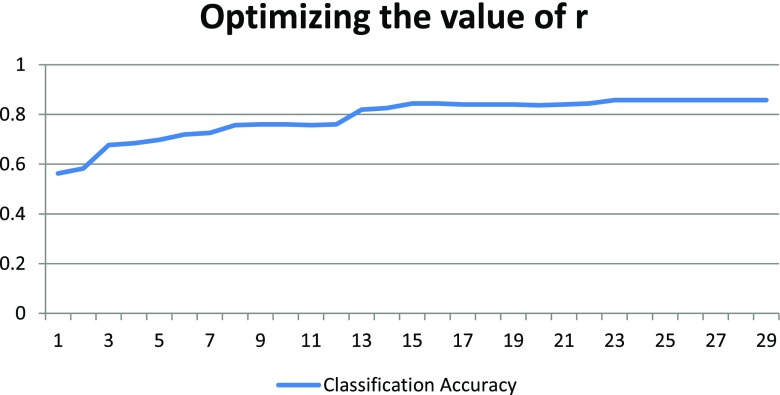
Selecting the optimal value of *r*. As can be seen the Classification Accuracy gradually increases by strating from value *r*= 1, after *r*= 23 the accuracy becomes stable

As explained in Section 4.2, we selected the value of *r* equal to 23. In order to select this optimal value of *r*, we executed the algorithm for a range of values for *r* starting from 1. After projecting the data to a single dimension, we used SMO at each value r from 1…29. At *r*= 23, we found stable as well as reliable classification results (Classification Accuracy equal to 85.57%) using a SMO classifier. The details of the classification results achieved for each value of r can be seen in Fig. 4.

Classification results for the SMO classifier as well as for 4 other classifiers, i.e., BN, KNN, DT, and ADTree (using 10-runs 10-FCV) on the transformed data by using the Scalable-LDA approach can be found in the first column of Table 1.

The overall results were improved when compared to SVM (on average the accuracy was 96%).

**Table 1:** Classification accuracy (%) for SMO, BN, KNN, DT and ADTree

	Scalable-LDA	PCA-LDA
SMO	85.57 ± 5.63%	55.85 ± 7.84%
BN	98.61 ± 1.97%	51.27 ± 1.06%
KNN	97.64 ± 2.64%	49.53 ± 8.63%
DT	98.61 ± 1.97%	51.27 ± 1.06%
ADTree	98.30 ± 2.17%	52.36 ± 8.16%

In addition to classification accuracy, another commonly used evaluation metric is area under the ROC curve (AUC) (normally denoted as A_*z*_), which is used to explain the diagnostic ability of the classifier [22]. The values of A_*z*_ by using 10-FCV classification scheme for 5 classifiers: SMO, BN, KNN, DT, ADTree are 0.853, 0.975, 0.972, 0.975, and 0.985, respectively, which indicates the stability of the classifiers (excluding SMO).

### About projected data

**Fig. 5:**
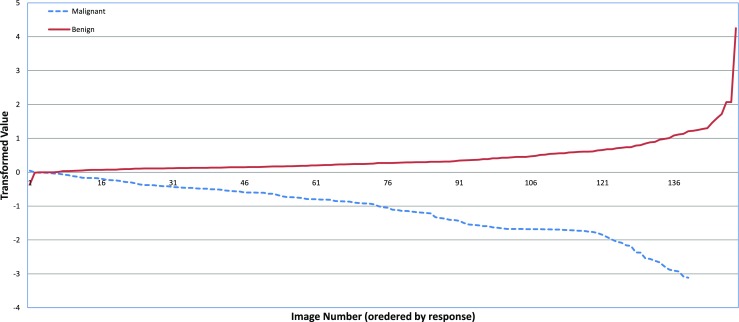
Transformed micro-calcification data. Almost all the malignant images have been transformed to the negative axis, whereas the benign images have been transformed to the positive axis. Only a small amount of data has been misclassified that have been shown at the left hand side of each curve for the benign and malignant class

While examining the projected data, we found that the values ≥ 0 are classified as benign, whereas the values < 0 are classified as malignant, which is shown in Fig. 5. The result in Fig. 5 cover 139 images from the malignant and 149 images from the benign class. It can also be clearly seen from Fig. 5 that this linear transformation of the segmented micro-calcification data is linearly separable.

**Fig. 6:**
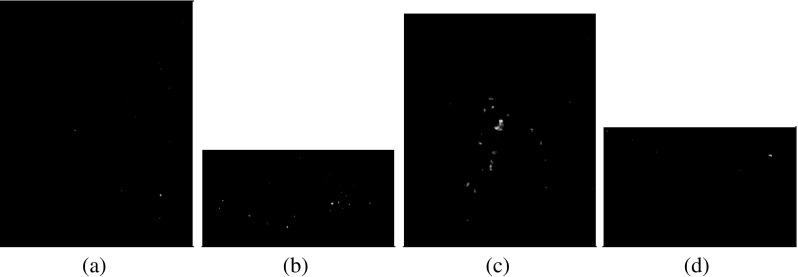
Sample RoIs from the benign class that have been correctly classified: The projected values for the RoIs shown in **a**–**d** are 0.125, 0.416, 1.295, and 0.109, respectively

**Fig. 7:**
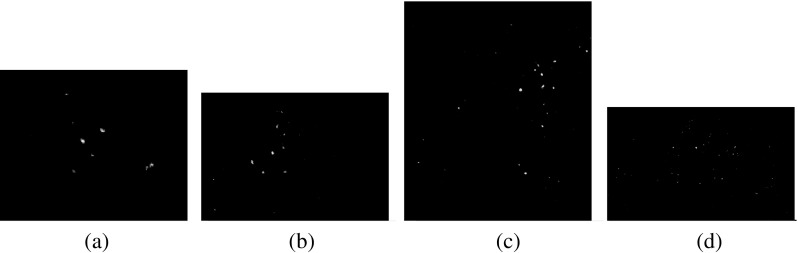
Sample RoIs from the malignant class that have been correctly classified: The projected values for the RoIs showed in the figure from **a**–**d** are − 0.207, − 0.607, − 2.517, and − 1.615, respectively

Figures 6 and 7 are showing sample cases (for both the benign and malignant classes) from the referenced dataset that have been correctly classified by using the developed approach. The projected value for each of these RoIs can also be seen in the figure’s captions.

## Discussion on misclassified data

**Fig. 8:**
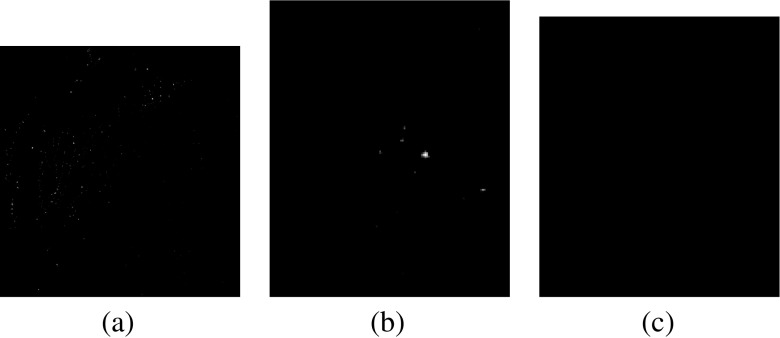
Three mis-classified RoIs found in the classification results of all the five classifiers (SMO, BN, KNN, DT, ADTree). **a** Misclassified benign RoI (projected value − 0.359), **b** Miscalssified malignant RoI (projected value: 0.046), **c** Misclassified malignant RoI (projected value: 0)

While observing the misclassified data, we observed characteristics of misclassified instances for all the classifiers used in our experiments (SMO, BN, KNN, DT and ADTree). We found 3 instances in all the classifiers (1 from the benign and 2 from the malignant class). Upon investigating the images from these misclassified data, the one benign image is very similar in appearance to the malignant images (having a dense cluster of micro-calcifications) and one malignant image has similarity with benign RoIs, whereas one of the malignant image have no detected micro-calcification. These three misclassified RoIs are shown in Fig. 8.

As stated in Section 1, several two stage approaches have also been proposed for LDA implementation [3, 4], which reduce the dimensionality of the data at the first stage before applying the actual LDA algorithm. We applied another version of LDA on our micro-calcification data for the benign and malignant classification that first transformed data to some low-dimensional space by using PCA before applying linear transformation of data (using LDA).

We used the Gram matrix (Appendix A) here for the computation of eigen vectors required for PCA. If D is representing a data matrix, then the Gram matrix for D is represented as *DD*^*T*^. The Gram matrix has been used in the literature for solving such eigenvalue problems and dimensionality reduction for large data sets [23, 24]. As our data matrix D is having dimension n×m and m ≫ n (for our dataset m = 10404612, n = 288), it is better to compute the eigen vectors of matrix *DD*^*T*^ instead of *D*^*T*^*D*. The relationship between the two matrices *D*^*T*^*D* and *DD*^*T*^ can be found in Appendix B. After computing the eigen vectors *ν* of *DD*^*T*^ we left multiply *ν* by *D*^*T*^ in order to get the eigen vectors of *D*^*T*^*D*. In order to consider matrix *DD*^*T*^, we already normalized the data matrix D to zero mean in order to consider *DD*^*T*^ as the covariance matrix of D.

**Fig. 9:**
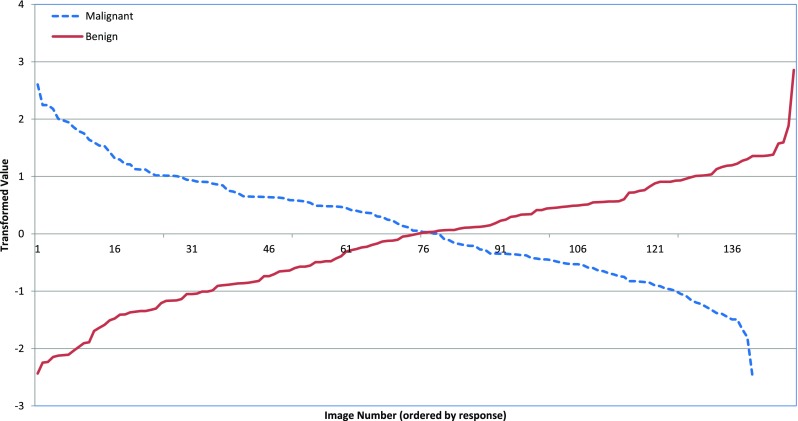
Transformed micro-calcification data by using the PCA-LDA approach. As can be seen, the data could not be classified correctly based on the transformed data. This is also in accordance with the results presented in column 2 of Table 1, according to which on average 52% of classification’s accuracy could be achieved from the transformed data by using the PCA-LDA approach

First we apply PCA on our data by setting the intermediate dimension to 10. We tried to set this dimension to 23, in order to be consistent with the Scalable-LDA approach (Section 4), but due to the limitations posed by the current memory we had to set the intermediate dimensions to 10. It should be noted here that we used the Gram matrix here for computing the eigen vectors, in order to cope with the memory requirements in computing eigen vectors of *D*^*T*^*D*. After that we transformed this reduced dimensional data to another linear space by using LDA. We used the LDA algorithm available with sklearn which is a machine learning library available with Python. The python version that was used in this experiment was version 2.7.0. The results of transforming the data matrix D to a linear dimension after applying applying LDA can be seen in Fig. 9. As can be clearly seen from the figure, the data is not linearly separable. We used the same classifiers (SMO, BN, KNN, DT, and ADT) and the average classification accuracy was 52% which is far less than the accuracy achieved by the developed approach (i.e. 96%), with details provided in Table 1. The results of the proposed scalable-LDA approach with setting the intermediate dimension r equal to 10 resulted in classification accuracy of 75% using the SMO classifier, whereas the results for BN, KNN,DT and ADTree classifiers were similar as in Table 1.

### Comparative analysis

Multiple methods have been developed in the past for the purpose of classifying benign and malignant micro-calcifications [25, 26]. Some of these approaches rely on the features extracted from individual calcifications [27, 28], whereas some focused on extracting global features from clusters of calcifications [29–32]. Ma et al. [27], suggested the roughness of the individual micro-calcification as a discriminatory property for classifying benign and malignant micro-calcifications. Similarly, other shape features (measures of compactness, moments and Fourier descriptors) have also been studied in the past [28] to measure the roughness of the contours of calcifications and then used as a measure of classifying the benign and malignant micro-calcifications. In a work related to extracting cluster level features of micro-calcifications [29], 23 features were extracted from micro-calcifications. The features were categorized into three general types as: intensity statistics, shape features and linear structure features. They also used balanced learning and optimized decision making for the classification of the micro-calcification clusters. Final results used two classifiers (Artificial Neural Network (ANN) and Support Vector Machine (SVM)) for classifying benign and malignant micro-calcifications. In addition, topological models have also been studied in the past for modeling and classification of micro-calcifications [30–32]. Both fixed-scale [32] and multi-scale approaches [30, 31] have been proposed in the past for classifying the micro-calcifications with promising results. In addition, the topological models are also useful for radiologists/doctors for visual interpretation of the underlying micro-calcification’s structure.

Apart from the methods developed for manual feature extraction (both for individual calcifications or cluster level feature extraction), to our knowledge no method has been developed so far that focussed on the automatic features extraction through dimensionality reduction techniques for classifying the benign and malignant micro-calcifications. The work in this paper presents a novel application of feature extraction through dimensionality reduction techniques for classifying the micro-calcifications as benign or malignant. The results are comparable with other state-of-the-art approaches developed to solve the same problem. The detailed comparison of the proposed technique with the state-of-the-art approaches is shown in Table 2.

**Table 2:** Results comparison with existing state-of-the-art approaches for the classification of benign and malignant micro-calcification

Author	Database	No. of cases	Accuracy (%)	A_*z*_
Shen et al. [28]	Unspecified	18	100	–
Ma et al. [27]	DDSM	183	80	0.76
Ren et al. [29]	DDSM	150	–	avg (SVM & ANN) 0.94
Chen et al. [30]	DDSM	300	86	0.90
Strange et al. [31]	DDSM	300	80	0.82
Suhail et al. [32]	DDSM	129	91	–
Scalable−LDA	DDSM	288	96	0.95

For the Scalable-LDA approach, average results for the 5 classifiers has been reported

The performance measures used for the comparison are Classification Accuracy and area under the ROC curve (A_*z*_). The comparison has been made with 6 existing methods. For the current work, average values for the classification accuracy and A_*z*_ are reported for the 5 classifiers used in the experiments.

## Future work

The basic purpose of this research was to develop a method to classify binary images which contain benign or malignant micro-calcifications. In the future, we will try to use this scalable-LDA approach for the classification of normal and abnormal mammographic images from the DDSM database: to convert the mammographic images into some binary representation before applying this scalable-LDA approach.

Although the results are satisfactory (average accuracy 96%), other methods exist in the literature for making LDA/PCA scalable [3, 4, 33], and we will do some comparative work by applying these approaches to the same dataset.

## Conclusions

We executed a two-stage LDA approach on binary data representing benign and malignant micro-calcifications. The idea was to project the data onto a low dimensional space and then use the resulting information to classify the data into two classes, but undersampling caused problems. By implementing a two-stage LDA approach, we achieved results that are comparable to state-of-the art approaches. The current method also presents a way to encode binary micro-calcification data as a single value. We achieved an accuracy of 96% on average for 5 classifiers, with the best performance at 98.6% by applying a scalable LDA approach for the classifications of benign and malignant micro-calcification. We compared the results with applying PCA-LDA on the same data, indicating a clear difference between the two approaches (Table 1, column 2).

### Data availability

The data that support the findings of this study are available from Prof. Reyer Zwiggelaar, Aberystwyth University but restrictions apply to the availability of these data, which were used under license for the current study, and so are not publicly available. Data are however available from the authors upon reasonable request and with permission of Prof. Reyer Zwiggelaar, Aberystwyth University.

## Appendix A: Gram matrix

Consider m vectors $\in \mathbb {R}^{n}$ [*x*_1_....*x*_*m*_]. The Gram matrix for this data collection is the m × m matrix G with elements *G*_*i**j*_ = ${x_{i}^{T}}x_{j}$. The matrix can be expressed as matrix X = [*x*_1_,...,*x*_*m*_], as

$$G = X^{T}X = \left[\begin{array}{c} {x_{1}}^{T}\\ {\vdots} \\ {x_{m}}^{T} \end{array}\right] \left[ x_{1} {\ldots} x_{m} \right] $$

## Appendix B: Eigenvectors from Gram matrix

Let A be the matrix having dimension m×n. Gram matrix of A can be computed as *A**A*^*T*^. If *ν* and *λ* is representing eigen values and eigen vector of Gram matrix *A**A*^*T*^, then

$$AA^{T} \nu = \lambda \nu $$

Left multiply both sides by *A*^*T*^, we get

$$A^{T} (AA^{T} \nu) = A^{T} (\lambda \nu) $$

Then by re-arranging terms:

$$(A^{T}A) (A^{T} \nu) = \lambda (A^{T} \nu) $$

Therefore, if *ν* is an eigenvector of *A**A*^*T*^, then *A*^*T*^*ν* is an eigenvector of *A*^*T*^*A* with the same eigenvalue *λ*.
